# Novel circNSD2 promotes proliferation and testosterone synthesis while inhibiting apoptosis in bovine Leydig cells

**DOI:** 10.3389/fvets.2026.1932131

**Published:** 2026-09-14

**Authors:** Xiang Meng, Tianxiang Meng, Xinqi Zhou, Sihua Jin, Yunhai Zhang, Ning Song, Hongyu Liu

**Affiliations:** 1 Anhui Province Key Laboratory of Local Livestock and Poultry Genetic Resource Conservation and Germplasm Innovation, College of Animal Science and Technology, Anhui Agricultural University, Hefei, China; 2 National Livestock and Poultry Regional Gene Bank (Anhui), Anhui Agricultural University, Hefei, China; 3 Institute of Beef Cattle Industry, Anhui Agricultural University, Hefei, China

**Keywords:** apoptosis, bovine Leydig cells, circNSD2, proliferation, testosterone synthesis

## Abstract

Leydig cells (LCs) are the primary cells responsible for testosterone synthesis in male animals, and their steroidogenic function is crucial for testicular development and spermatogenesis in bulls. Circular RNAs (circRNAs) play important roles in various biological processes and reproductive development; however, their functions in bovine LCs remain largely unexplored. Our previous whole-transcriptome sequencing of calf and adult bovine testes identified a differentially expressed circRNA, circ_0027599 (designated circNSD2 after its host gene *NSD2*), that was highly expressed in adult testes. To investigate its function, circNSD2 was overexpressed in bovine LCs, and its effects on cell proliferation, apoptosis, and testosterone synthesis were assessed. The results showed that circNSD2 overexpression significantly enhanced cell viability, increased the proportion of cells in the S phase, and upregulated the expression of proliferation-related genes (proliferating cell nuclear antigen [*PCNA*] and *CDK2*). Concurrently, circNSD2 overexpression significantly reduced the number of apoptotic cells, downregulated the expression of pro-apoptotic genes (*BAX* and *Caspase3*), and upregulated the anti-apoptotic gene (*Bcl2*). Furthermore, circNSD2 overexpression promoted testosterone secretion and increased the expression of key steroidogenic genes (*STAR*, *CYP11A1*, *CYP17A1*, and *HSD17B3*). In conclusion, circNSD2 promotes proliferation, inhibits apoptosis, and enhances testosterone synthesis and secretion in bovine LCs. These findings reveal a potential regulatory role of circNSD2 in bovine testicular development and provide a theoretical foundation and molecular target for improving reproductive performance in breeding bulls.

## 1 Introduction

The reproductive potential of male livestock is a critical economic trait in modern animal husbandry. The reproductive performance of breeding bulls directly determines herd productivity and the efficiency of genetic improvement, and is therefore essential for the sustainable development of the industry (1). Wandong is an indigenous yellow cattle breed in eastern Anhui, China, exhibiting desirable traits, including strong roughage tolerance and disease resistance. However, breeding bulls of this breed commonly exhibit low libido and suboptimal semen quality, which severely hinder conservation and breeding programs (2). Recent studies have reported the regulatory roles of circular RNAs (circRNAs) in male reproductive development (3), however, research on circNSD2 remains limited. Therefore, investigating the regulatory mechanisms of functional genes in testicular development is essential for enhancing the reproductive value and economic benefits of Wandong cattle (4).

The testis is the primary male reproductive organ responsible for producing male gametes and secretes androgens (5). Leydig cells (LCs), located in the interstitial compartment between the seminiferous tubules, are the main cells responsible for testosterone synthesis and secretion (6). Testosterone, a key androgen, is essential for sexual development, maintenance of reproductive function, spermatogenesis, and regulation of reproductive performance in bulls. Its synthesis involves a complex multi-step enzymatic process regulated by luteinizing hormone (LH) (7). After binding to its membrane receptor on LCs, LH activates adenylate cyclase, which converts ATP into cAMP and subsequently upregulates the expression of the steroidogenic acute regulatory protein (*STAR*) (8). *STAR* mediates cholesterol transport from the outer mitochondrial membrane to the inner mitochondrial membrane, which is the initial and rate-limiting step in testosterone synthesis (9). Subsequently, cholesterol is converted to pregnenolone by cytochrome P450 family 11 subfamily A member 1 (*CYP11A1*). Pregnenolone is then sequentially catalyzed by cytochrome P450 family 17 subfamily A member 1 (*CYP17A1*), 3β-hydroxysteroid dehydrogenase (*3β-HSD*), and 17β-hydroxysteroid dehydrogenase type 3 (*HSD17B3*) in the smooth endoplasmic reticulum, ultimately yielding testosterone (10). Among these, *STAR*, *CYP11A1*, *CYP17A1*, and *HSD17B3* are key marker genes for testosterone synthesis, and their expression levels directly determine the testosterone-synthesizing capacity of LCs (11, 12). Abnormal expression of these genes can lead to decreased testosterone levels and impaired reproductive function.

CircRNAs are a class of closed-loop endogenous non-coding RNAs formed by the back-splicing of precursor mRNAs. They lack 5′ caps and 3′ poly(A) tails and are characterized by stable expression and resistance to degradation by exonucleases (13, 47). CircRNAs function primarily as miRNA sponges, regulators of RNA-binding proteins, or transcriptional modulators and participate in diverse biological processes, including cell proliferation, apoptosis, and differentiation (14, 46). *NSD2*, which encodes a histone methyltransferase, has been implicated in cell proliferation, differentiation, and tumorigenesis (15). However, the biological function of circNSD2 in LCs remains unclear. Our previous whole-transcriptome sequencing experiment revealed high circNSD2 expression in the testes of adult Wandong bulls, suggesting its potential involvement in regulating bovine LC function (16).

Therefore, this study aimed to investigate the effects of circNSD2 overexpression in bovine LCs. The findings provide a theoretical basis for further elucidating the molecular mechanisms of circNSD2, improving the reproductive performance of Wandong breeding bulls, and optimizing conservation strategies for local breeds.

## 2 Materials and methods

### 2.1 Ethics statement

Testis samples were collected from three healthy 3-year-old adult Wandong bulls at Fengyang Daming Agricultural Animal Husbandry Technology Development Co., Ltd. The samples were cleaned with iodophor, 70% ethanol, and PBS containing 2% penicillin/streptomycin and were immediately transported on ice to the laboratory for primary LC isolation. All animal procedures were approved by the Animal Ethics Committee of Anhui Agricultural University (SYXK2021-009).

### 2.2 Culture of LCs

Primary bovine LCs were isolated from the testicular tissue of the three Wandong bulls and subsequently established and cryopreserved in our laboratory, as previously described (17). The tissue was digested with collagenase type P (Merck Millipore, Darmstadt, Germany) and centrifuged at 210 ×*g* for 45 min. The supernatant was discarded, and the upper tissue pellet was filtered through 100- and 200-mesh steel sieves. The filtered tissue was washed with Dulbecco’s Modified Eagle Medium (DMEM)/F12 (SH30023, Hyclone, Logan, UT, USA), and centrifuged at 210 ×*g* for 15 min. LCs were further purified using the density gradient centrifugation. The gradient consisted of six phases of Percoll (P8370, Solarbio, Beijing, China) at concentrations of 5, 30, 40, 50, 60, and 70%. The filtered cell suspension was layered onto the gradient and centrifuged at 1,000 ×*g* for 30 min at 18 °C. The cell band located between the 40 and 50% Percoll layers was collected, washed with DMEM/F12 medium, and centrifuged at 210 ×*g* for 15 min at 34 °C. The resulting cells were resuspended in DMEM/F12 medium supplemented with 10% fetal bovine serum. When 80% confluence was reached, LCs were identified via 3β-HSD staining. Cells were cultured in DMEM/F12 supplemented with 10% fetal bovine serum and 1% penicillin/streptomycin at 37 °C in a 5% CO₂ incubator.

For all experiments, LCs at passages 2–4 were used. Cells were seeded in different culture plates at the following densities: 1 × 10^6^ cells/well in 6-well plates for RNA and protein extraction and cell cycle and apoptosis analyses; 4 × 10^5^ cells/well in 6-well plates for the 5-ethynyl-2′-deoxyuridine (EdU) assay; 5 × 10^3^ cells/well in 96-well plates for the CCK-8 assay; and 2 × 10^5^ cells/well in 24-well plates for testosterone measurement. After seeding, cells were cultured for 24 h until they reached 60–70% confluence prior to transfection.

### 2.3 Identification of circRNA

Analysis of previous whole-transcriptome sequencing data (NCBI database BioProject PRJNA760322) from calf and adult bovine testes identified a differentially expressed circRNA, novel_circ_0027599, which was named circNSD2 based on its host gene, *NSD2* (16). To verify its circular structure, total RNA was treated with RNase R (Beyotime, Shanghai, China) at a final concentration of 2 U /μg at 37 °C for 20 min, and incubated at 70 °C for 10 min. After treatment, RT-PCR was performed using *GAPDH* as a linear control. The amplification products were analyzed by electrophoresis and then sent to Beijing Liuhe BGI Genomics Co., Ltd. for sequencing to verify the back-splicing site. The full-length sequence of circNSD2 is shown in Supplementary Tables S1 and S2.

### 2.4 Cell transfection

The full-length circNSD2 sequence was amplified and cloned into the pEGFP-N1 vector to generate the circNSD2 overexpression plasmid (pEGFP-N1-circNSD2). The empty pEGFP-N1 vector was used as a negative control to eliminate any potential confounding effects of the vector backbone on cell function. All constructs were synthesized by Sangon Biotech (Shanghai, China) and verified by Sanger sequencing. For transfection, LCs were seeded into appropriate culture plates and cultured to 60–70% confluence. Transfections were performed using Lipofectamine 3,000 (Invitrogen, USA) according to the manufacturer’s protocol. For 6-well plates, 2.5 μg of plasmid DNA and 5 μL of Lipofectamine 3,000 were used per well. At 24 h post-transfection, cells were harvested for RNA extraction. At 48 h post-transfection, cells were collected for protein extraction, cell cycle analysis, apoptosis assays, and EdU assays, while culture supernatants were collected for testosterone quantification.

### 2.5 RNA extraction and quantitative RT-PCR

Reverse transcription was performed in a 20 μL reaction containing 2 μg of total RNA using TRUEscript RT Master Mix (Aidlab, China) according to the manufacturer’s instructions. RT-qPCR was performed on a CFX96 Real-Time PCR System (Bio-Rad, USA) in a 10 μL reaction composed of 5 μL 2 × SYBR Green qPCR Master Mix (Aidlab, China), 0.4 μL each of forward and reverse primers (10 μM), 1 μL cDNA template, and 3.2 μL of RNase-free water. The thermal cycling conditions were as follows: 95 °C for 2 min, followed by 40 cycles of 95 °C for 15 s, 60 °C for 20 s, and 72 °C for 30 s. Melting curve analysis was conducted from 65 °C to 95 °C in 0.5 °C increments (5 s per step) to confirm amplification specificity. All samples were analyzed in triplicate, and each experiment was independently repeated three times. *GAPDH* was used as the internal control, and relative gene expression levels were calculated using the 2^−ΔΔCt^ method (18). All primer sequences are listed in Supplementary Table S3.

### 2.6 Western blotting

Treated LCs were collected and lysed on ice using High-Efficiency Rapid RIPA Lysis Buffer (Solarbio) to extract proteins. The protein concentration was determined using a bicinchoninic acid (BCA) assay kit. After separation by 10% SDS-PAGE, protein samples were transferred to a polyvinylidene fluoride membrane (PVDF; IPVHO0005, Merck, Germany) and blocked with 5% skim milk at room temperature (18–25 °C) for 1 h. The membranes were incubated with primary antibodies overnight at 4 °C. After three washes with TBST, the membranes were incubated with secondary antibodies at room temperature for 2 h. Finally, protein signals were detected using an ECL Chemiluminescent Substrate Kit (BL502B, Biosharp, China) and the Alliance Q9 Advanced imaging system. Grayscale values were analyzed using ImageJ (v1.8.0), with *β*-actin used as the internal control. The specific antibodies and dilution ratios are listed in Supplementary Table S4.

### 2.7 Cell viability assay

Cells were seeded in 96-well plates, and cell viability was assessed at 0, 24, 48, 72, and 96 h using the CCK-8 kit according to the manufacturer’s instructions. Absorbance at 450 nm was measured using the TECAN Spark^®^(Switzerland).

### 2.8 Apoptosis and cell cycle analysis

Cells were seeded in 6-well plates at a density of 1 × 10^6^ cells per well. After transfection, the cells were harvested, washed with PBS, and analyzed by flow cytometry using an Annexin V-FITC/PI Apoptosis Detection Kit (KeyGen, Jiangsu, China) to detect apoptosis in the LCs. The cells were treated with Annexin V-FITC and propidium iodide and incubated in the dark for 15 min. Binding buffer was then added, and apoptosis was measured using a flow cytometry (BD Accuri C6 Plus, USA). For cell cycle analysis, cells were fixed in 70% ethanol at 4 °C overnight, washed with PBS, and incubated in the dark at 37 °C with RNase A/PI solution at a ratio of 1:9 for 30 min before detection using an Accuri C6 flow cytometer. The data were analyzed using FlowJo v10.

### 2.9 EdU incorporation assay

Cells were seeded in 6-well plates at a density of 4 × 10^5^ cells per well. After transfection, cell proliferation was assessed using the BeyoClick EdU Kit (C0085L Beyotime). The nuclei of proliferating cells were stained brown with EdU and observed under a phase-contrast microscope (Axio Vert, Zeiss). Quantitative analysis was performed using ImageJ software.

### 2.10 Enzyme-linked immunosorbent assay (ELISA)

LC culture supernatants were collected 48 h after transfection and centrifuged at 3,000 ×*g* for 10 min. Testosterone levels were then measured using a Bovine Testosterone ELISA Kit (Kexing, Shanghai, China), and absorbance was detected at 450 nm using a microplate reader (TECAN, Switzerland). Testosterone concentrations were calculated using a standard curve.

### 2.11 Data analysis

Statistical analysis was performed using GraphPad Prism v10 (GraphPad Software, USA), and an independent samples *t*-test was applied. *p* < 0.05 was considered statistically significant.

## 3 Results

### 3.1 Identification of circRNAs

Novel_circ_0027599 was identified as a 468-bp circRNA formed by back-splicing of the NSD2 gene located on chromosome 6 (Figure 1A). This circRNA is hereinafter referred to as circNSD2. To validate circNSD2, its back-splicing site was amplified using divergent primers and confirmed by Sanger sequencing (Figure 1B). RNase R is a ribonuclease that efficiently digests linear RNA molecules but not circRNAs. RNA extracted from cells was treated with or without RNase R. RT-PCR results showed that both linear *GAPDH* mRNA and circNSD2 were amplified in the untreated group, whereas only circNSD2 was amplified in the RNase R-treated group (Figure 1C). These results indicate that circNSD2 is a stable circRNA.

**Figure 1:**
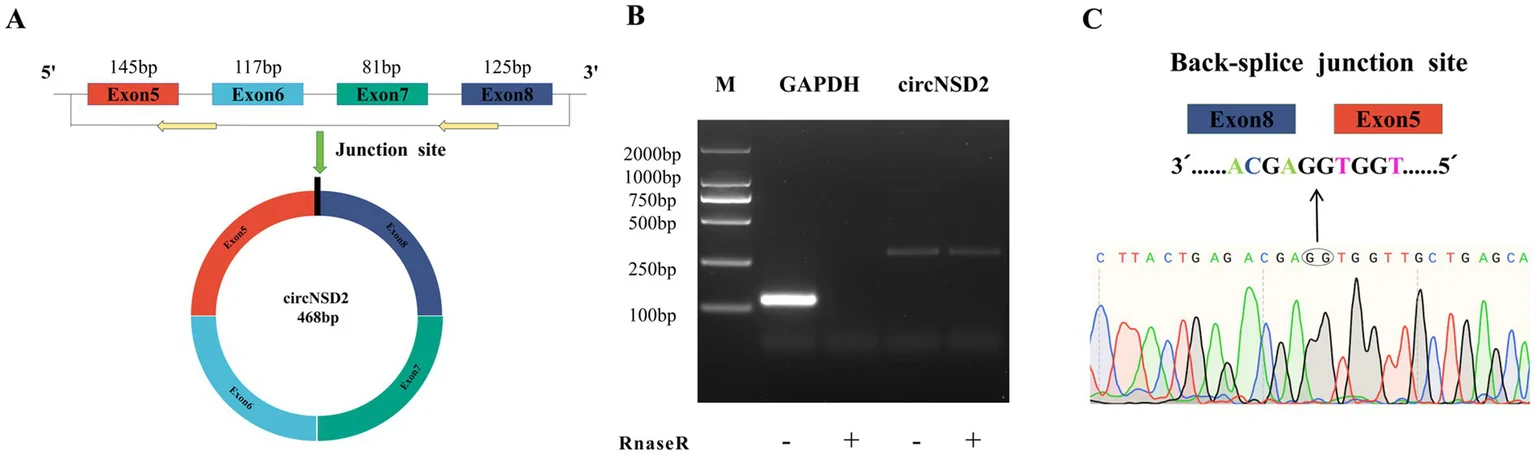
Identification of the circular structure of circNSD2. **(A)** The reverse splicing site of circNSD2 was identified by Sanger sequencing. **(B)** RT-PCR analysis of RNA treated with RNase R or left untreated. **(C)** circNSD2 is formed by the circularization of exons 5, 6, 7, and 8 of the source gene.

### 3.2 circNSD2 overexpression promotes cell proliferation and cell cycle progression

After the circular structure of circNSD2 was confirmed, its function in LCs was investigated. RT-qPCR was used to assess the overexpression efficiency of the circNSD2 overexpression vector in bovine LCs. The results showed that circNSD2 expression was significantly higher in the overexpression group than in the control group (Figure 2A, *p* < 0.001). Subsequent RT-qPCR and western blotting showed that circNSD2 overexpression significantly increased the mRNA and protein expression levels of the proliferation-related genes proliferating cell nuclear antigen (*PCNA*) and *CDK2* (Figures 2B,C; Supplementary Figure S1, *p* < 0.01). Similarly, CCK-8 and EdU assays demonstrated that the proliferative capacity of LCs was significantly enhanced after circNSD2 overexpression (Figures 2D,E, *p* < 0.01). Furthermore, flow cytometric analysis of the cell cycle revealed that the proportions of cells in the G0/G1 and G2/M phases did not change significantly after circNSD2 overexpression, whereas the proportion of cells in the S phase increased significantly (Figure 2F, *p* < 0.001).

**Figure 2:**
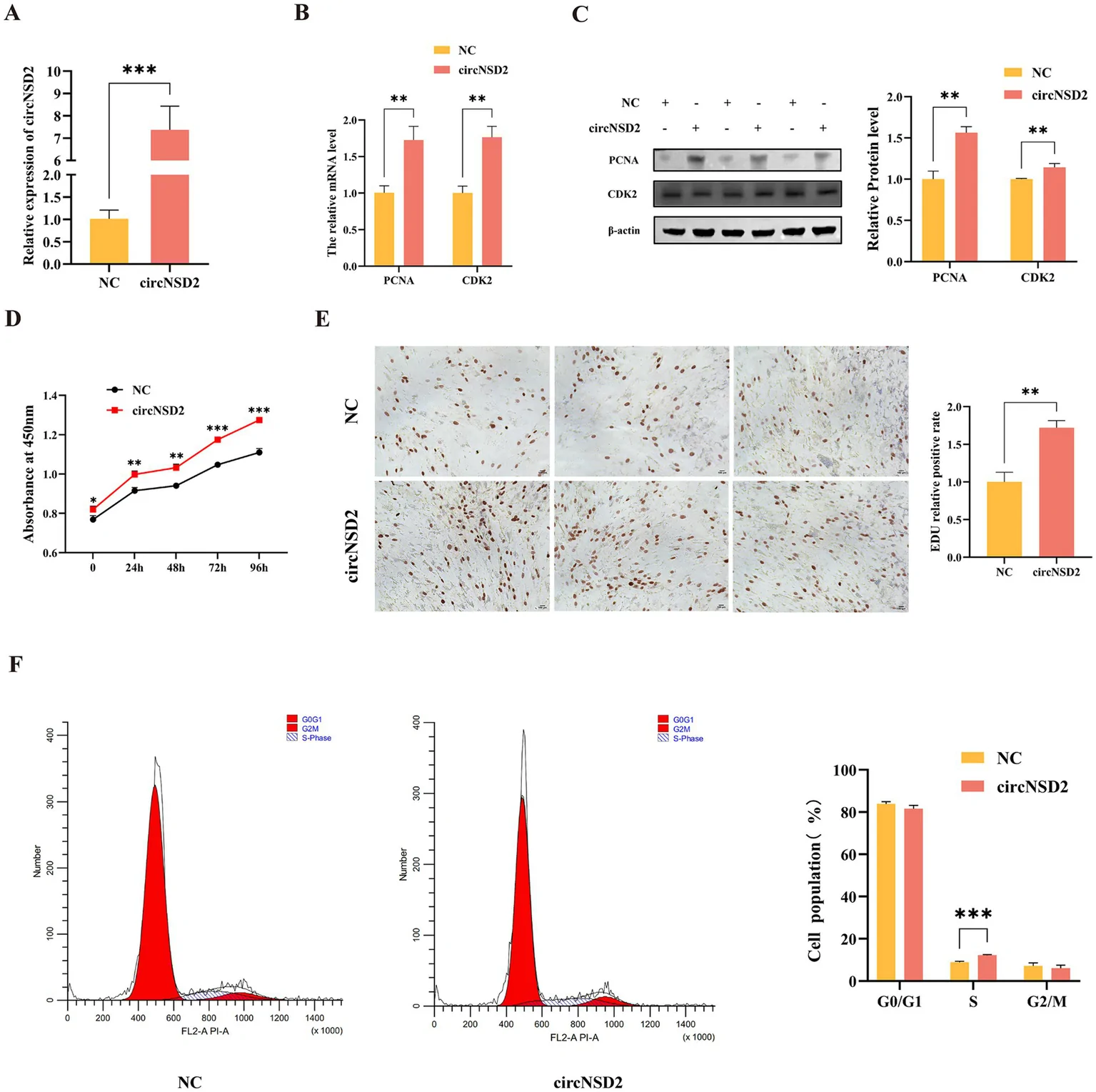
Overexpression of circNSD2 promotes the proliferation of Leydig cells. **(A,B)** RT-qPCR analysis of circNSD2 overexpression efficiency and mRNA expression levels of proliferation-related genes. **(C)** Western blot analysis of protein expression levels of proliferation-related genes. **(D)** Cell viability assay using the CCK-8 assay. **(E)** Cell proliferation assay using EdU staining. **(F)** Cell cycle distribution analysis by flow cytometry.

### 3.3 circNSD2 overexpression inhibits apoptosis

To investigate the role of circNSD2 in LC apoptosis, the expression levels of apoptosis-related genes were examined after circNSD2 overexpression using RT-qPCR and western blotting. The results showed that circNSD2 overexpression significantly decreased the mRNA expression levels of the pro-apoptotic markers *Caspase3* and *BAX* (*p* < 0.05), whereas it significantly increased the mRNA expression level of the anti-apoptotic gene *Bcl2* (Figure 3A, *p* < 0.01). At the protein level, Caspase 3 expression was significantly reduced (*p* < 0.05), whereas *BAX* expression showed no significant change. In contrast, *Bcl2* expression was significantly elevated (Figure 3B; Supplementary Figure S2, *p* < 0.05). Subsequently, apoptosis was detected by flow cytometry, and the results showed that circNSD2 overexpression significantly reduced the total apoptosis rate of LCs (Figure 3C, *p* < 0.001).

**Figure 3:**
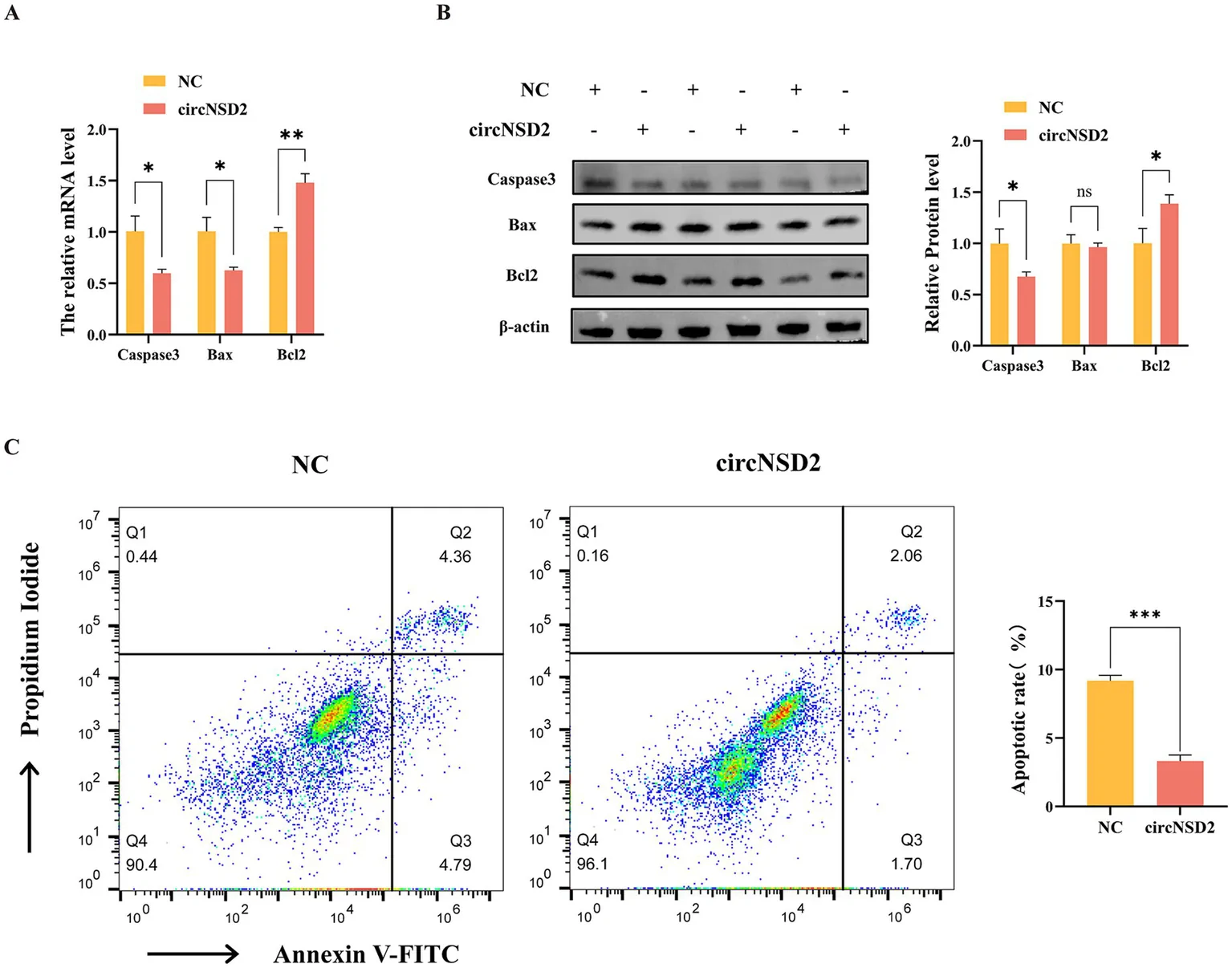
Overexpression of circNSD2 inhibits apoptosis in LCs. **(A)** mRNA expression levels of apoptosis-related genes were detected by RT-qPCR. **(B)** Protein expression levels of apoptosis-related genes were detected by Western blot. **(C)** Total cell apoptosis rate was detected by flow cytometry.

### 3.4 circNSD2 overexpression promotes testosterone synthesis

To further investigate the potential role of circNSD2 in testosterone synthesis in LCs, the expression of testosterone synthesis-related genes and testosterone secretion levels after circNSD2 overexpression was examined using RT-qPCR, western blotting, and ELISA. RT-qPCR and western blotting showed that circNSD2 overexpression significantly increased the mRNA and protein expression levels of the testosterone synthesis-related genes *STAR*, *CYP11A1*, *CYP17A1*, and *HSD17B3* (Figures 4A,B; Supplementary Figure S3, *p* < 0.01). ELISA results showed that circNSD2 overexpression significantly increased testosterone concentration in the LC culture supernatant (Figure 4C, *p* < 0.05).

**Figure 4:**
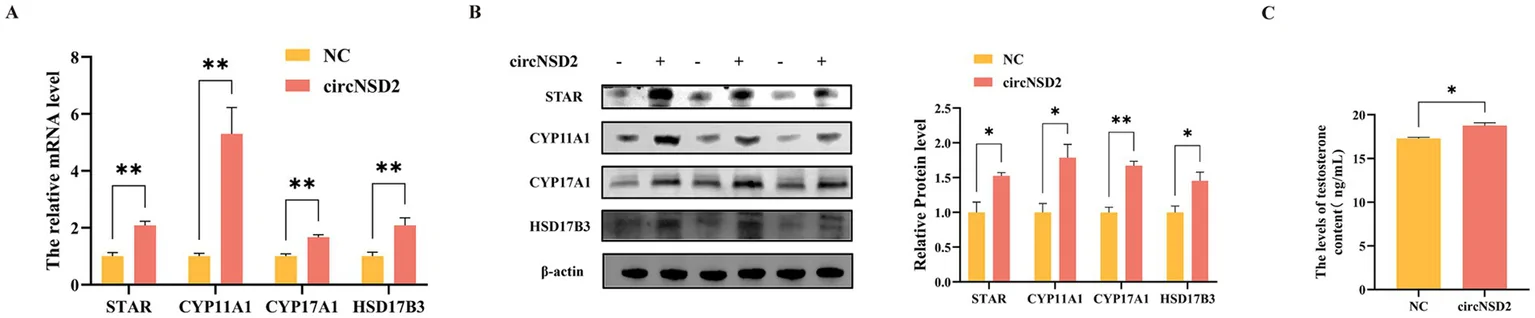
Overexpression of circNSD2 promotes testosterone synthesis in LCs. **(A)** mRNA expression levels of genes related to testosterone synthesis were detected by RT-qPCR. **(B)** Protein expression levels of genes related to testosterone synthesis were detected by Western blot. **(C)** Testosterone levels were detected by ELISA.

## 4 Discussion

Testosterone biosynthesis is a complex enzymatic process in which *STAR* mediates cholesterol transport into the mitochondria (19). Subsequently, *CYP11A1* catalyzes the conversion of cholesterol to pregnenolone, whereas *CYP17A1* further converts pregnenolone to 17α-hydroxypregnenolone, which is then converted into the testosterone precursors dehydroepiandrosterone and androstenedione (20, 44). Finally, *HSD17B3* converts androstenedione into testosterone (21, 45). The expression levels of these four proteins directly determine the testosterone-synthesizing capacity of LCs. Knockdown of *Bmal1* reduces the expression of key testosterone synthesis-related genes in mouse LCs, including *STAR*, *CYP11A1* and *3β-HSD*, and may affect testosterone secretion and apoptosis by regulating the PI3K/AKT signaling pathway (22). In rat LCs, Ad-ADM alleviates the LPS-induced reduction in LC survival and suppresses apoptosis by inhibiting TGF-β1 production through the Hippo signaling pathway, thereby restoring steroid synthesis *in vitro* (23). Other studies have shown that spermidine attenuates Poly(I:C)-induced immune responses, oxidative stress, and apoptosis, alleviates the downregulation of *STAR*, *CYP11A1*, and *CYP17A1*, and restores testosterone production in yak LCs (24). Based on previous studies, *STAR*, *CYP11A1*, *CYP17A1*, and *HSD17B3* were selected as key marker genes to investigate the regulatory role of circNSD2 in testosterone synthesis in bovine LCs (25). The results showed that circNSD2 overexpression significantly upregulated the mRNA and protein expression levels of these four marker genes and promoted testosterone secretion, indicating that circNSD2 positively regulates testosterone synthesis in bovine LCs.

In addition to steroid synthesis, the proliferative capacity and apoptotic homeostasis of LCs are critical for maintaining testicular function (26). Impaired proliferation or excessive apoptosis can lead to LC depletion and reduced testosterone output, ultimately resulting in decreased fertility or infertility (27). *PCNA* is a cofactor for DNA polymerase *δ* and is indispensable for DNA replication and repair, and its expression level is directly correlated with the proportion of cells in the S phase (28). In rat LCs, endogenous LH deficiency significantly reduces the levels of *Cyclin D3* and *PCNA*, which are associated with cell proliferation, while simultaneously decreasing the expression of *Igf1* mRNA, which is crucial for LC proliferation and development (29). Isorhamnetin enhances the G₁/S transition mediated by the cyclin E/CDK2 complex by reducing inhibitory phosphorylation at Tyr-15 of *CDK2* and upregulating CDK2 protein expression, thereby inducing cells to enter S phase. Simultaneously, it downregulates cyclin A expression, ultimately arresting cells in the S phase (30). Cyclin-dependent kinase 2 (*CDK2*) drives the G_1_/S transition by forming complexes with cyclin E and cyclin A (31). Therefore, this study selected *PCNA* and *CDK2* as key markers for evaluating the proliferative capacity of LCs. Although this study confirmed the pro-proliferative effect of circNSD2 using CCK-8, EdU, cell cycle analysis, and PCNA/CDK2 expression, a detailed exploration of the G1/S regulatory machinery (e.g., cyclin E family members) was beyond the scope of this work. Further investigation of the interaction between circNSD2 and specific cell cycle regulators is warranted to fully elucidate the underlying molecular mechanism.

Apoptosis is another fundamental process that regulates cellular homeostasis, and the intrinsic mitochondrial apoptotic pathway is primarily regulated by the BCL2 family (32). Upon activation, the pro-apoptotic member *BAX* increases outer mitochondrial membrane permeability, induces cytochrome c release, and subsequently activates the caspase cascade (33). In contrast, the anti-apoptotic protein *Bcl2* maintains mitochondrial integrity by binding to *BAX* and preventing its oligomerization (34). *Caspase3* is the common final effector protease in both intrinsic and extrinsic apoptotic pathways, and its activation irreversibly leads to cell death (35). In mouse LCs, alcohol induces apoptosis by activating specific apoptotic pathways, upregulating *BAX* and *Caspase3*, and simultaneously downregulating *Bcl2* (36). Selenomethionine significantly increases the Bcl2/Bax ratio in sheep LCs under heat exposure by downregulating the pro-apoptotic gene *BAX* and upregulating the anti-apoptotic gene *Bcl2*, thereby inhibiting apoptosis and effectively alleviating heat stress-induced phosphorylation-mediated activation of p38MAPK and HSPB1 in sheep LCs (37). Therefore, based on previous studies on LCs, *BAX*, *Bcl2*, and *Caspase3* were selected as the key apoptotic marker genes. This study found that circNSD2 overexpression significantly reduced the mRNA levels of *BAX* and *Caspase3*, increased *Bcl2* expression, and significantly reduced the overall apoptosis rate of bovine LCs. Notably, although the *BAX* protein levels showed a downward trend, no significant difference was observed. Owing to post-transcriptional or translational regulation, such as protein degradation, mRNA expression may not necessarily correlate with final protein expression (38). Therefore, the anti-apoptotic effect of circNSD2 is likely achieved primarily through the upregulation of *Bcl2* and inhibition of *Caspase3* rather than through direct downregulation of *BAX* protein levels.

CircRNAs are widely expressed in the male reproductive system and are involved in testis development, spermatogenesis, and regulation of germ cell function (39). By binding to let-7i, circSMC1B regulates the *HMGA1*/*NR6A1* axis to promote the proliferation of bovine male germline stem cells and participate in bovine spermatogenesis (40). circPAN3 promotes the proliferation of immature porcine Sertoli cells and inhibits their apoptosis by regulating the expression of proliferation- and apoptosis-related genes (41). *NSD2*, a histone methyltransferase, participates in cell proliferation, differentiation, and tumorigenesis (42). Studies have shown that *NSD2* protein is highly enriched in spermatocytes and round spermatids, and *NSD2* deficiency leads to increased germ cell apoptosis, elevated sperm abnormalities, and ultimately impaired male fertility (43). In this study, circNSD2 overexpression promoted proliferation, inhibited apoptosis, upregulated testosterone synthesis-related genes, and increased testosterone secretion in bovine LCs (Figure 5), thereby advancing the functional characterization of circRNAs in these cells and broadening the known biological roles of NSD2-related non-coding RNAs. However, although this study conducted circNSD2 overexpression experiments, loss-of-function experiments are still needed to determine the function of circNSD2. In addition, the molecular mechanisms by which circNSD2 regulates LC proliferation, apoptosis, and testosterone synthesis remain to be investigated. In future studies, addressing these limitations will strengthen the understanding of circNSD2 in testicular function.

**Figure 5:**
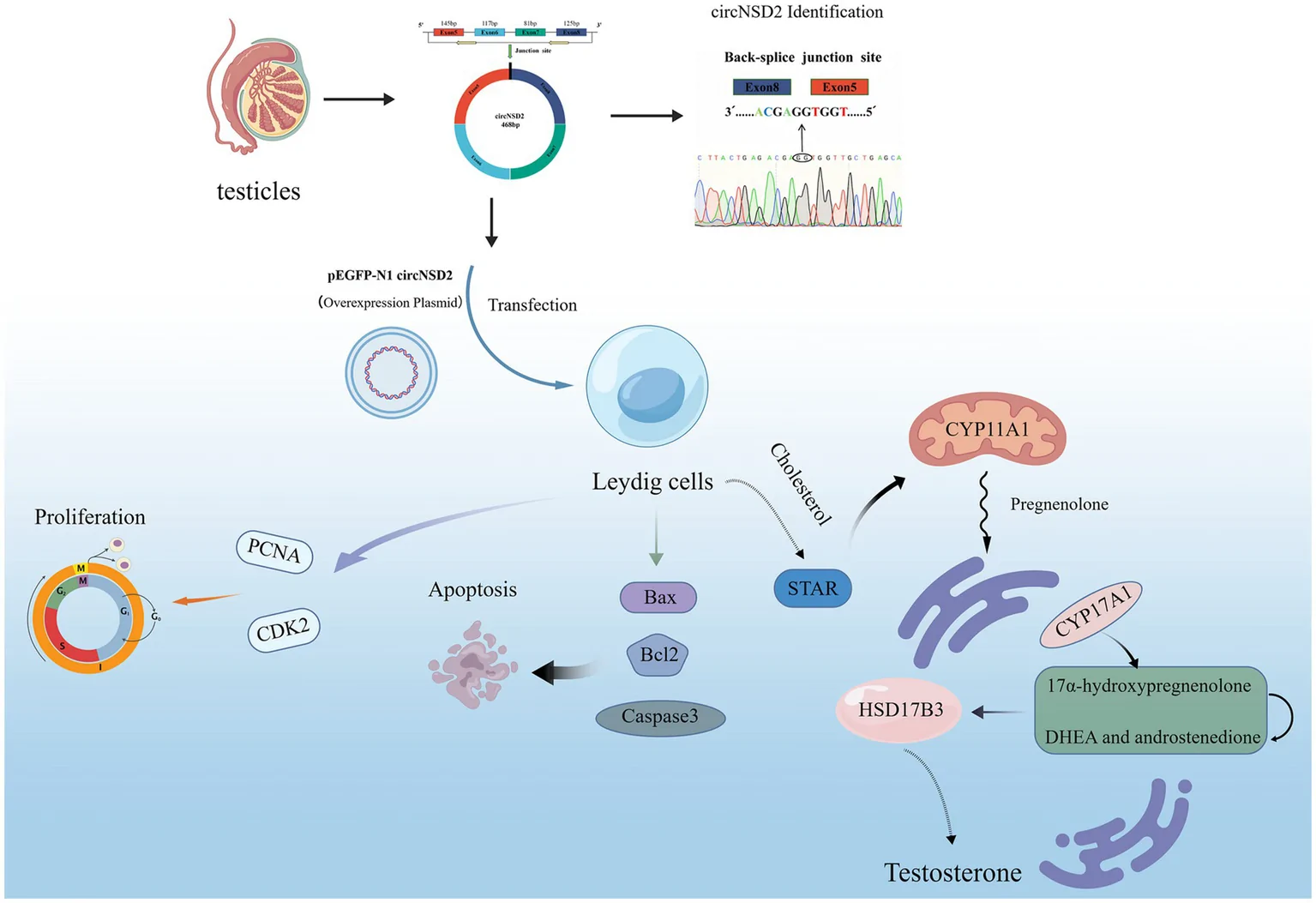
Schematic diagram illustrating the regulatory mechanisms of circNSD2 in LCs regarding proliferation, apoptosis, and testosterone synthesis.

## 5 Conclusion

This study provides preliminary evidence for the positive regulatory role of circNSD2 in the proliferation, apoptosis, and testosterone synthesis of bovine LCs. These findings provide a theoretical foundation for further investigation of the molecular mechanisms of circNSD2 in bull reproductive regulation and identify a new candidate target for improving bull reproductive performance and optimizing reproductive management.

## Data Availability

The transcriptome data presented in the study have been deposited in the NCBI BioProject repository, accession number PRJNA760322. The data are publicly available at: https://www.ncbi.nlm.nih.gov/bioproject/PRJNA760322.
