# Supplementary material for: Novel circNSD2 promotes proliferation and testosterone synthesis while inhibiting apoptosis in bovine Leydig cells
Source: Front Vet Sci. 2026 Sep 14;13:1932131. doi: 10.3389/fvets.2026.1932131 (PMC13616769; doi:10.3389/fvets.2026.1932131)
Supplement: Supplementary file 1 [file Data_Sheet_1.pdf]

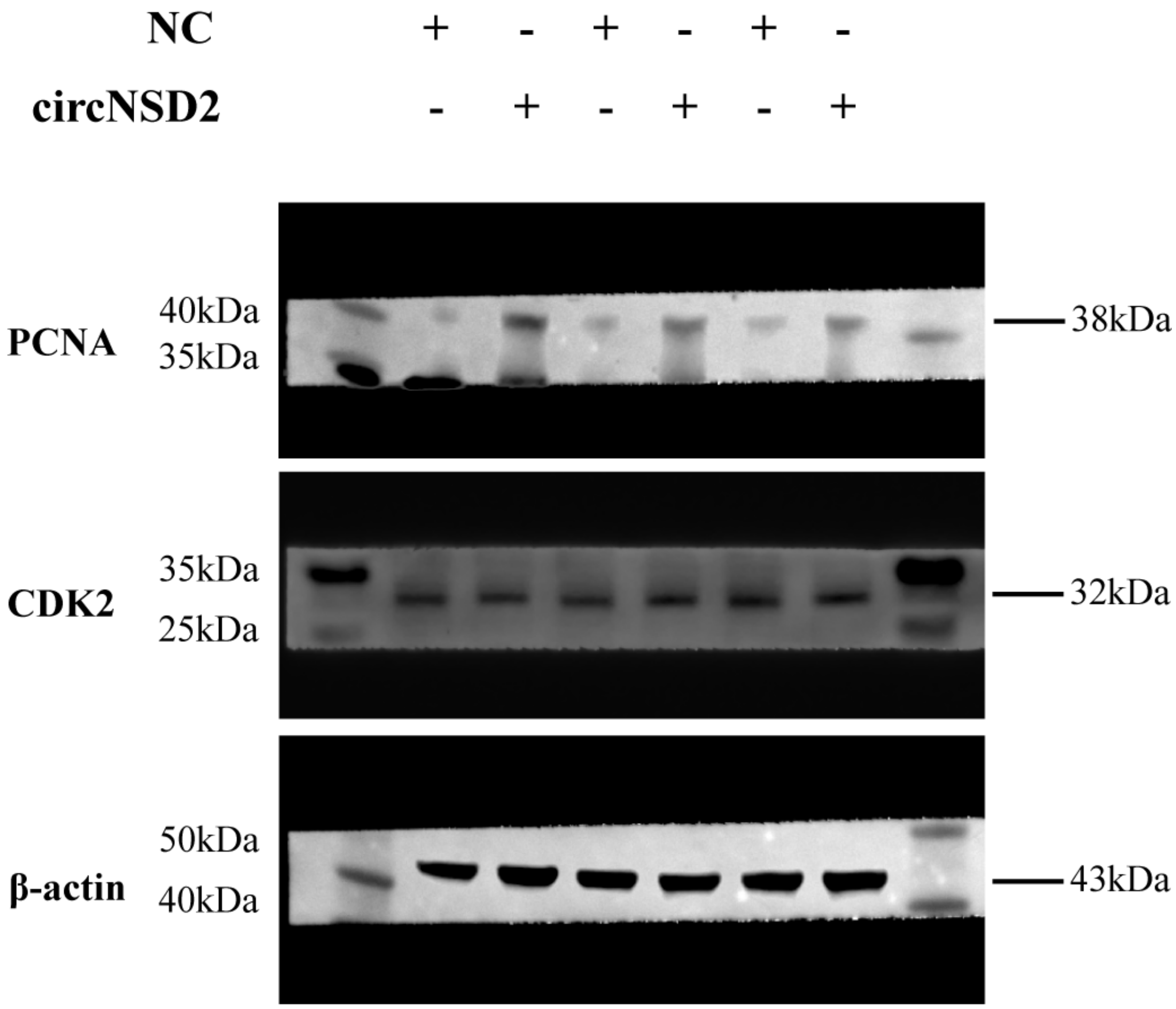

Supplementary Fig. S1. The full western blotting image of Fig. 2C. The protein abundance of PCNA and CDK2 in bovine Leydig cells transfected with the overexpression of circNSD2 and NC for 48 h.

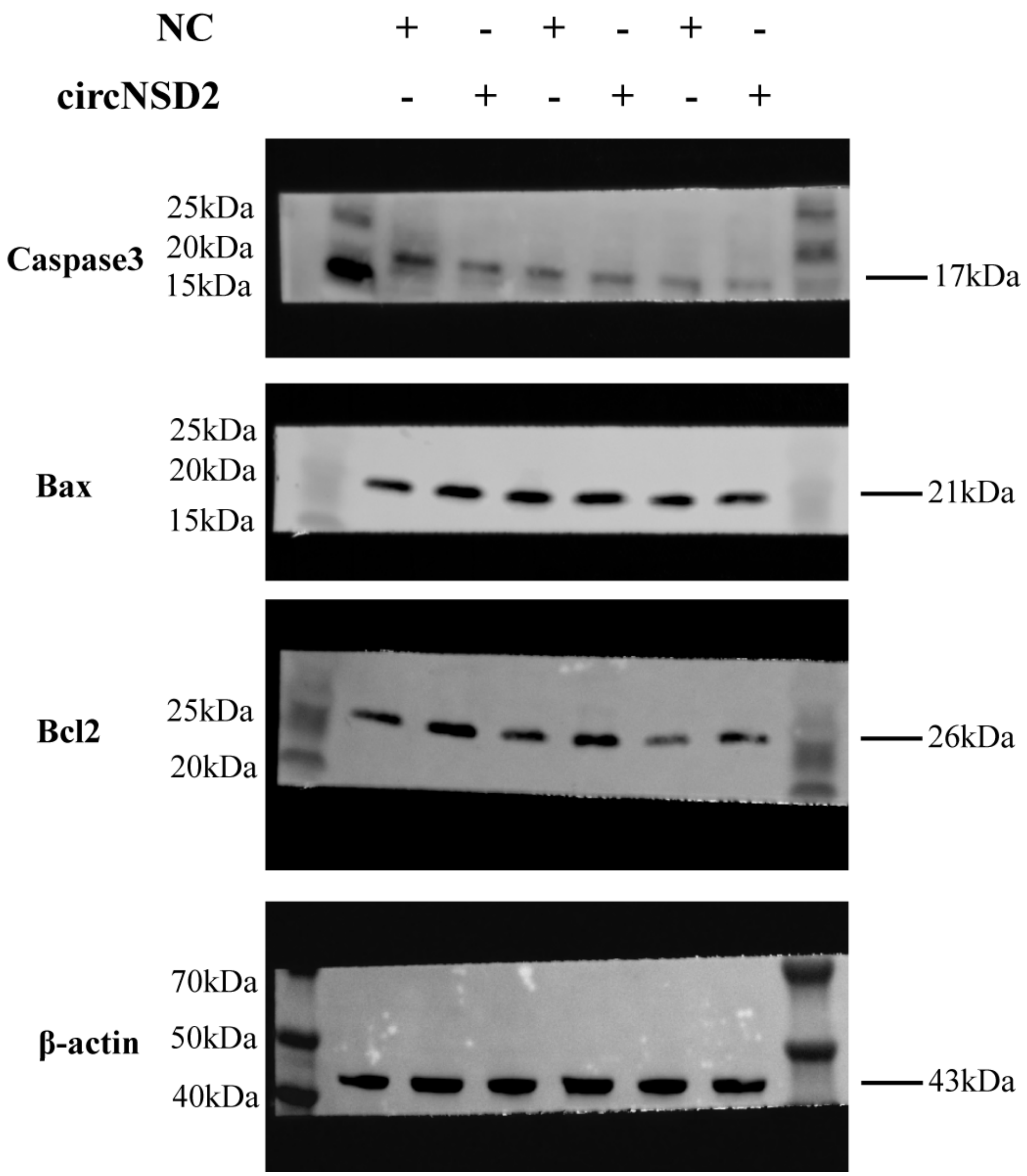

Supplementary Fig. S2. The full western blotting image of Fig. 3B. The protein abundance of Caspase3, Bax and Bcl2 in bovine Leydig cells transfected with the overexpression of circNSD2 and NC for 48 h.

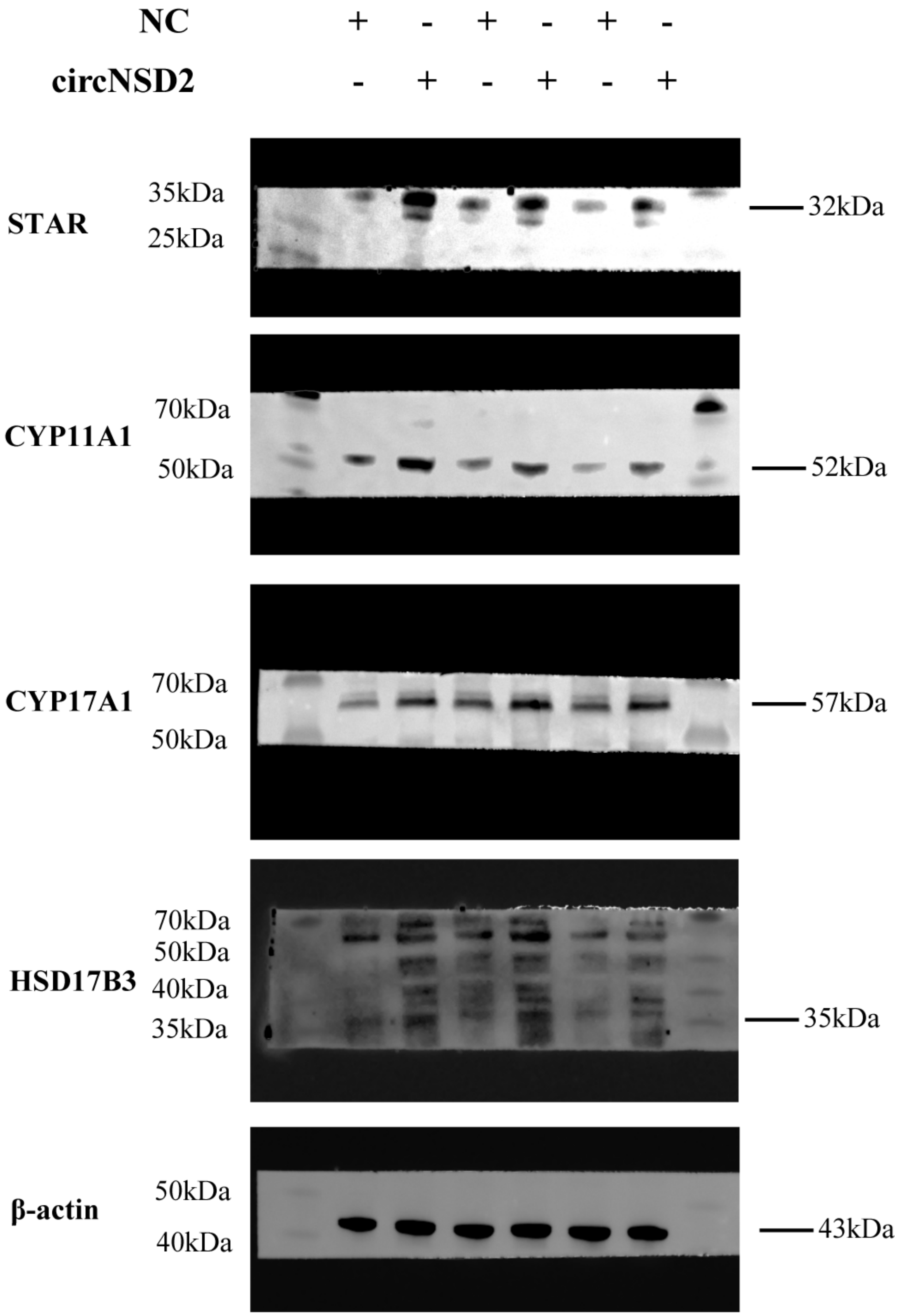

Supplementary Fig. S3. The full western blotting image of Fig. 4B. The protein abundance of STAR, CYP11A1, CYP11A1 and HSD17B3 in bovine Leydig cells transfected with the overexpression of circNSD2 and NC for 48 h.
