# Supplementary material for: Novel circNSD2 promotes proliferation and testosterone synthesis while inhibiting apoptosis in bovine Leydig cells
Source: Front Vet Sci. 2026 Sep 14;13:1932131. doi: 10.3389/fvets.2026.1932131 (PMC13616769; doi:10.3389/fvets.2026.1932131)
Supplement: Supplementary file 2 [file Table_1.DOCX]

Table S1. Linear sequence of the circNSD2 precursor.

GTGGTTGCTGAGCACCCGGACGCCTCTGCCGAGGAGATTGAGGAGCTGCTCGCGTCGCAGTGGAACATGCTCAACGAGAAGCAGAAGGCGCGCTATCACACCAAGTTTGCGCTCGTGGCTCCGCCTCAGTCCGAGGAAGACTCTGTAACTTAACTTAATGGGAAGAAAAGGAGCCACACGAAGAGGACGCAGGGCCTGCGCGGAGACGCTGAAGTTGAGGAGGCCCCCAGGAAGAGGCTCAGGACTGACAGGCACGGCCTGCGGAAAGAGAGACGATCACTGACAAAACAGCCCGCACGACCTCTTGCAAGGCCCTTGAGGCAGCGTCCTCACTGAAGAGCCAGGCACCACGAAACACCTGTCTGATGCCTGCAAGCCCCTGAAGAAGCGCCACCGGGCCCCCGCAGCGGCCGCCACCACCCTCACCTTTAGCAAAAGCTCATCTCCTTCCGCGTCCTTAACTGAGAACGAG

Note: Base sequences highlighted in red, blue, green and gray correspond to exons 5, 6, 7 and 8 of bovine NSD2 gene, respectively.

Table S2. Mature circular sequence of circNSD2.

AGAGAGACGATCACTGACAAAACAGCCCGCACGACCTCTTGCAAGGCCCTTGAGGCAGCGTCCTCACTGAAGAGCCAGGCACCACGAAACACCTGTCTGATGCCTGCAAGCCCCTGAAGAAGCGCCACCGGGCCCCCGCAGCGGCCGCCACCACCCTCACCTTTAGCAAAAGCTCATCTCCTTCCGCGTCCTTAACTGAGAACGAGGTGGTTGCTGAGCACCCGGACGCCTCTGCCGAGGAGATTGAGGAGCTGCTCGCGTCGCAGTGGAACATGCTCAACGAGAAGCAGAAGGCGCGCTATCACACCAAGTTTGCGCTCGTGGCTCCGCCTCAGTCCGAGGAAGACTCTGTAACTTAACTTAATGGGAAGAAAAGGAGCCACACGAAGAGGACGCAGGGCCTGCGCGGAGACGCTGAAGTTGAGGAGGCCCCCAGGAAGAGGCTCAGGACTGACAGGCACGGCCTGCGGAA

Note: Base sequences highlighted in green, gray, red and blue correspond to exons 7, 8, 5 and 6 of bovine NSD2 gene, respectively.

Table S3. Primer sequences used for RT-qPCR.

| **Gene** |  | **Primer sequences (5′-3′)** | **Product size**  **(bp）** | **GenBank accession number** |
| --- | --- | --- | --- | --- |
| PCNA | F: | GAACCTCACCAGCATGTCCA | 86 | NM_001034494.1 |
|  | R: | ACGTGTCCGCGTTATCTTCA |  |  |
| CDK2 | F: | TCTTTGCTGAGATGGTGACCC | 201 | NM_001014934.1 |
|  | R: | CATCTTCATCCAGGGGAGGC |  |  |
| Bax | F: | TCTGACGGCAACTTCAACTG | 112 | NM_173894.1 |
|  | R: | CCATGATGGTCCTGATCAACTC |  |  |
| Bcl2 | F: | TGGATGACCGAGTACCTGAA | 133 | NM_001166486.1 |
|  | R: | GAGACAGCCAGGAGAAATCAAA |  |  |
| Caspase3 | F: | CCTGAATGTCAGCCCGTAAA | 108 | NM_001077840.1 |
|  | R: | TCTTCTGCAAAGGATGGATGAG |  |  |
| STAR | F: | CGGAAGCTCCTACAGACA | 157 | NM_174189.3 |
|  | R: | CTGAGAACCTAGGAGAGAGC |  |  |
| CYP11A1 | F: | TTCAACCTCATCCTGACGCC | 204 | NM_176644.2 |
|  | R: | GTGCAAGAGGTGTGGACTGA |  |  |
| CYP17A1 | F: | GCCAAGGTGAATGCAGACAA | 240 | NM_174304.3 |
|  | R: | GTTCCGGTCACTGATGGTTG |  |  |
| HSD17B3 | F: | GCTGTCATTCTGAGCCTGAT | 100 | NM_001076439.2 |
|  | R: | TGGCATTCTTCTTGAGGTAGTC |  |  |
| GAPDH | F: | GCATCCCTGAGACAAGATGGTG | 113 | NM_001034034.2 |
|  | R: | CATTGATGGCGACGATGTCCAC |  |  |
| circNSD2 | F: | GCCACCACCCTCACCTTTAG | 279 |  |
|  | R: | CCTCCTCAACTTCAGCGTCTC |  |  |

Table S4. Antibodies used for Western blot.

| **Antibody** | **Company** | **Cat NO** | **Dilution** | **Host** |
| --- | --- | --- | --- | --- |
| PCNA | Proteintech | 10205-2-AP | 1:5000 | Rabbit |
| CDK2 | Proteintech | 10122-1-AP | 1:10000 | Rabbit |
| Bax | Proteintech | 50599-2-lg | 1: 10000 | Rabbit |
| Bcl2 | Proteintech | 12789-1-AP | 1:10000 | Rabbit |
| Caspase3 | Proteintech | 19677-1-AP | 1:2000 | Rabbit |
| STAR | Songon | D223242 | 1:1000 | Rabbit |
| CYP11A1 | Proteintech | 13363-1-AP | 1:1000 | Rabbit |
| CYP17A1 | Bioss | bs-3853R | 1:2000 | Rabbit |
| HSD17B3 | Abcam | Ab126228 | 1:3000 | Rabbit |
| β-actin | Cwbio | CW0096M | 1:5000 | Mouse |
